# Does sourdough bread provide clinically relevant health benefits?

**DOI:** 10.3389/fnut.2023.1230043

**Published:** 2023-07-20

**Authors:** Vera D’Amico, Michael Gänzle, Lisa Call, Benjamin Zwirzitz, Heinrich Grausgruber, Stefano D’Amico, Fred Brouns

**Affiliations:** ^1^Department of Food Science and Technology, BOKU–University of Natural Resources and Life Sciences, Vienna, Austria; ^2^Department of Agricultural, Food and Nutritional Science, University of Alberta, Edmonton, AB, Canada; ^3^Department of Crop Sciences, BOKU–University of Natural Resources and Life Sciences, Tulln, Austria; ^4^Institute for Animal Nutrition and Feed, AGES–Austrian Agency for Health and Food Safety, Vienna, Austria; ^5^Department of Human Biology, School for Nutrition and Translational Research in Metabolism, Maastricht University, Maastricht, Netherlands

**Keywords:** sourdough fermentation, cereals, gastrointestinal disorders, immunogenic proteins, FODMAPs, health benefits, *in vivo* /*in vitro*/human studies

## Abstract

During the last decade, scientific interest in and consumer attention to sourdough fermentation in bread making has increased. On the one hand, this technology may favorably impact product quality, including flavor and shelf-life of bakery products; on the other hand, some cereal components, especially in wheat and rye, which are known to cause adverse reactions in a small subset of the population, can be partially modified or degraded. The latter potentially reduces their harmful effects, but depends strongly on the composition of sourdough microbiota, processing conditions and the resulting acidification. Tolerability, nutritional composition, potential health effects and consumer acceptance of sourdough bread are often suggested to be superior compared to yeast-leavened bread. However, the advantages of sourdough fermentation claimed in many publications rely mostly on data from chemical and *in vitro* analyzes, which raises questions about the actual impact on human nutrition. This review focuses on grain components, which may cause adverse effects in humans and the effect of sourdough microbiota on their structure, quantity and biological properties. Furthermore, presumed benefits of secondary metabolites and reduction of contaminants are discussed. The benefits claimed deriving from *in vitro* and *in vivo* experiments will be evaluated across a broader spectrum in terms of clinically relevant effects on human health. Accordingly, this critical review aims to contribute to a better understanding of the extent to which sourdough bread may result in measurable health benefits in humans.

## Introduction

The application of sourdough fermentation for bakery products is increasing, especially in large-scale bread production. The microbiota of sourdough consists of lactic acid bacteria and yeasts, which is responsible for specific flavor and quality of the final products, including delayed staling and prevention of microbial spoilage. In addition, it is widely claimed that sourdough fermentation improves the nutritional profile of bread and reduces the content of components that may cause gastrointestinal complaints in susceptible individuals. These claims are predominantly based on *in vitro* studies that document the degradation of (potentially) harmful or anti-nutritive factors, or an altered digestibility of macronutrients and micronutrients (1–3). Effects on health inferred from *in vitro* observations, however, were not confirmed by randomized clinical trials. Probably other factors than sourdough fermentation *per se*, such as type of grain, flour coarseness and product structure play a determining role. Alternatively, effects of sourdough bread when measured *in vivo* may be too small to result in significant changes (4–7). A recent meta-analysis of studies investigating the effect of sourdough fermentation on the glycemic index (GI) of bread provided no convincing evidence of positive effects of sourdough bread on either reducing GI or improving glucose homeostasis (8).

To date, the limited and inconclusive clinical data on health benefits of sourdough bread also prevented the approval of health claims by regulatory agencies including the European Food Safety Agency (EFSA). Despite apparently favorable data, EFSA has not approved any of the requested sourdough benefit claims so far. For example, a health claim related to the reduction of post-prandial glycemic responses by high fiber rye sourdough bread was not approved because the supporting studies demonstrated a reduced glycemic response relative to glucose solutions but not to comparable bread produced without sourdough (9). Here we aim to review the current state of science and the potential difficulties that may underlie the discrepancy between assumed benefits of sourdough fermentation and the apparently insufficient substantiation of these benefits in clinical trials.

Much of the data presented in dossiers submitted to EFSA for approval of health claims are based on the study of ‘isolated factors’ *in vitro, ex vivo* and in animals. For a correct interpretation of data obtained from the variety of nutrition studies, one needs to understand all aspects of the food being tested. In the case of bread, the choice of the grain type, region and year of harvest, the degree of milling (fine or coarse), type of flour (wholemeal or refined flour) and the way the dough is being processed (conditions of the fermentation and baking process) influence its composition in terms of contents of nutrients and compounds that may cause intolerances in some individuals. Differences in these parameters may, in turn, affect digestion and absorption in the human body, exposure to cells and organs, and ultimately health. In this light, one may question what data from laboratory experiments mean with respect to the situation in which humans consume the tested food as part of their daily meals containing many other components.

When it is claimed that sourdough bread in comparison to straight dough bread with baker’s yeast as sole fermentation organism results in a ‘better nutritional profile’ or makes a bread ‘better digestible’, it is of crucial importance to define what these terms stand for. Dependent of the factor of measure (time or quantity) ‘better digestibility’ can relate to less time required for a component to be completely digested or that quantitatively more of the food is digested and consequently less undigested matter passes into the colon. Further, does a ‘better nutritional profile’ mean that the components that may cause adverse reactions are themselves reduced or that anti-nutritive factors are reduced; or does it mean that more nutrients are present. Further, the question arises if such changes really lead to measurable health benefits, which is the ultimate proof for claim approval. Depending on what we define as ‘digestibility’, the parameters to be studied can be very different, which will influence the conclusions regarding health benefits.

For components, which may cause adverse reactions, the degradation of anti-nutritive factors during sourdough fermentation or during proofing of straight dough bread can be quantified. This includes phytate, lectins, amylase-trypsin inhibitors (ATIs), rapidly fermentable non-absorbed carbohydrates (FODMAPs), and gluten (10–16). Similarly, one can measure the content of specific nutrients that have been synthesized by the fermentation medium or released from the food matrix in sourdough bread compared to straight dough bread, like vitamins, phenolic compounds and minerals. However, results from chemical analyzes or *in vitro* assays pointing to possible benefits for health are often not confirmed *in vivo*. Thus, a critical question arises: does enhanced digestion rate and quantity, or a favorable content of certain components, as measured *in vitro,* have a significant effect on human health in terms of measured clinical endpoints?

### Animal vs. *in vitro* gastrointestinal models

To use *in vitro* and animal data as potentially valid for the prediction of the human *in vivo* situation, the consumption, gastrointestinal, hormonal and metabolic factors need to be at least ‘very similar’ to the human situation. Although frequently utilized, rats and mice have major differences. In contrast, swine have a digestive system that is similar to that of humans. This allows blood samples to be taken and calculations to be made on the distribution of nutrients throughout the body as well as their uptake by various organs. Data from the multiple cannulated swine model correlate well with human *in vivo* data (17–19). A significant drawback is the required surgery and high degree of specialization required, which makes its frequent use for multiple food testing impossible. To overcome these limitations, *in vitro* gastrointestinal models have been developed for rapid screening of potential digestibility and prediction of responses that are expected to occur *in vivo*, such as GI.

In this respect, a mechanical continuous digestion model, which includes the compartments of the stomach, duodenum, jejunum and ileum (TIM-1) and colon (TIM-2), has been developed (20). The TIM models have been improved over the last decade and it has been demonstrated that they produce valid test–retest data (21) as well as a high correlation when compared to both human and multi-cannulated pig data. In addition, the INFOGEST consortium has developed and validated a more straightforward statistical model (22–27).

**Figure 1:**
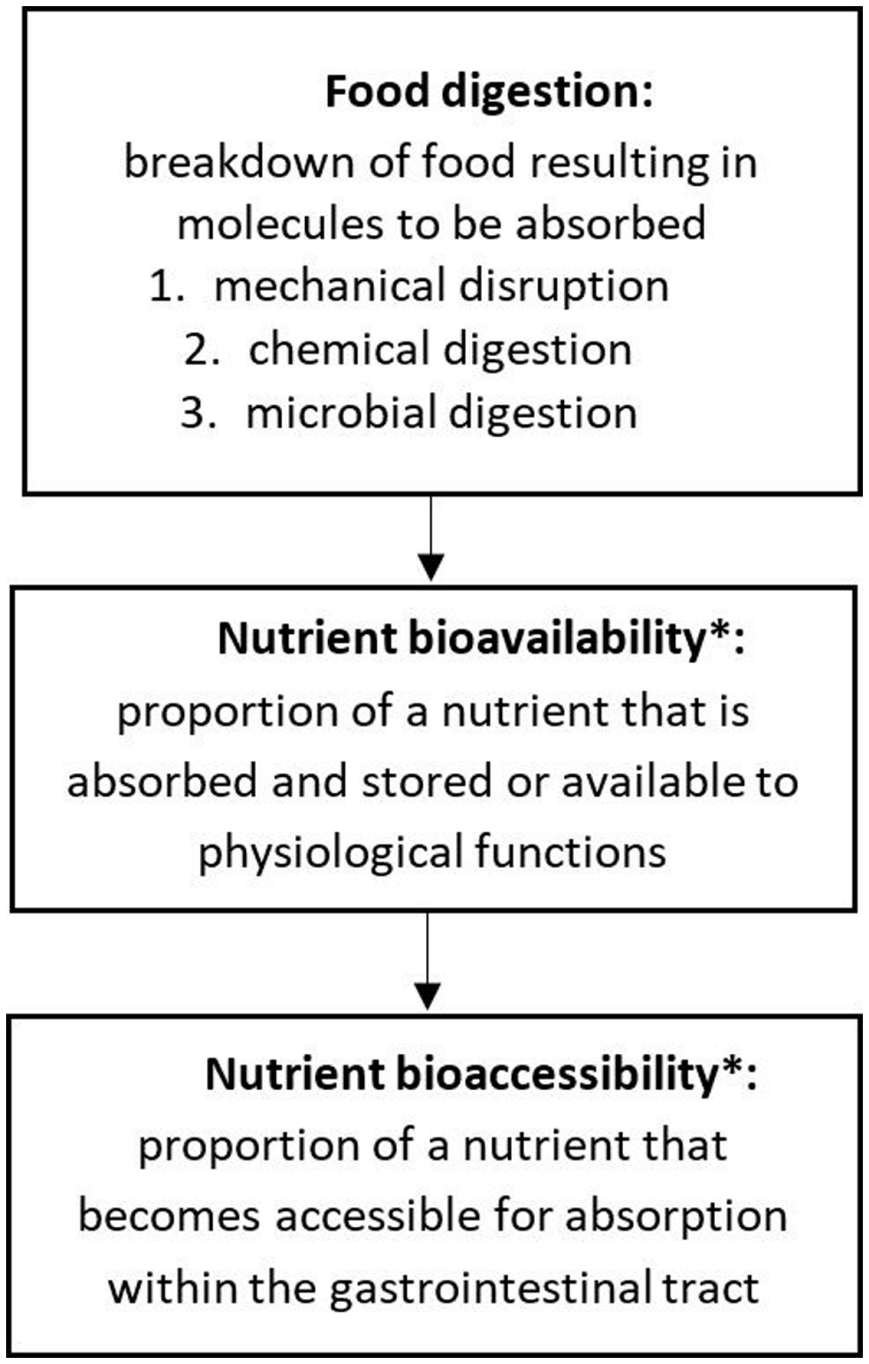
Definitions of the terms “food digestion,” “nutrient bioavailability” and “nutrient bioaccessibility.” *For detailed review see Dima et al. (4) and Fernandez-Garcia et al. (28).

Despite these promising developments, differences in the set-up and procedures of *in vitro* studies (e.g., duration of oral processing, type of enzymes used, exposure to the food sample) make direct quantitative comparisons of data across studies difficult. Furthermore, *in vitro* studies generally result in data on the nutrient content within the test digestion system. However, bio-accessibility is not the same as bio-availability and the two terms are often used interchangeably (Figure 1).

In the *in vitro* dynamic model, small molecules derived from digestion can pass through a microdialysis membrane. The rate and quantity of passage are often used as estimates of potential absorption in the human body. However, the *in vitro* model lacks the cells of the intestinal epithelium that may either consume or convert such molecules to other compounds. For example, small peptides are readily degraded by enterocyte proteases and extremely small quantities may pass into the blood. There they become subjected to hydrolysis by proteases, resulting in very short half-lives and concentrations too low to induce a biological effect (29–31). Accordingly, the presence of peptides in an *in vitro* digest has little relevance for their *in vivo* absorption and postabsorption bioactivity. As a result, before drawing appropriate conclusions about *in vivo* in humans, *in vitro* data must be critically examined.

## Adverse reaction to grain components

**Figure 2:**
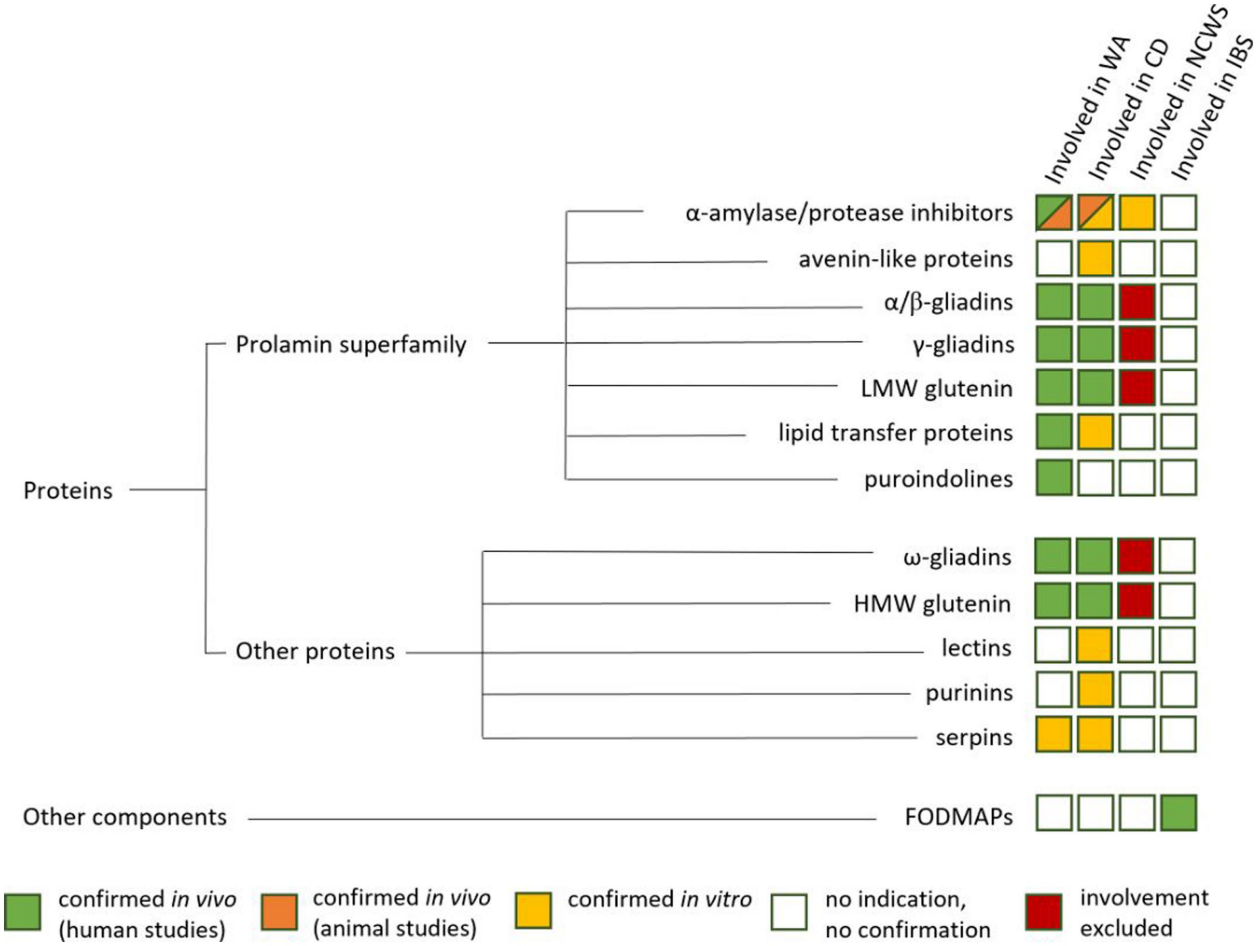
Proteins and other components and their involvement in wheat allergy (WA), Celiac Disease (CD), Non-Celiac Wheat Sensitivity (NCWS) and Irritable Bowle Sydrome (IBS) (34–45).

There are three main types of adverse reactions to grains, commonly known as: (1) celiac disease (CD), (2) wheat allergy, and (3) non-celiac wheat sensitivity (NCWS). Patients suffering from irritable bowel syndrome (IBS) show symptoms that strongly overlap with NCWS. Further, NCWS and IBS strongly overlap with fructose malabsorption (32). These disorders, except IBS, are summarized as gluten-related disorders (GRD) (33). An overview about components causing the mentioned disorders is given in Figure 2.

### Celiac disease

CD is an autoimmune disorder that is triggered by consuming gluten-containing grains produced from wheat, rye, barley, and triticale. Undigested gluten peptides may pass the luminal side of the intestinal epithelium and enter the lamina propria via the transcellular or paracellular route. Subsequently, the enzyme tissue transglutaminase (tTG) deaminates the peptides, and dendritic cells recognize specific amino acid sequences on the surface of the antigen (called epitopes), resulting in binding to antigen-presenting cells and the initiation of immune and inflammatory responses (46).

Globally, approximately 1% of the population is diagnosed with CD, but regional prevalence may vary (47). Intestinal biopsy specimen analysis revealed that 0.7% (0.5–0.9%) of the population worldwide was positive. However, the pooled global CD prevalence was 1.4% (1.1–1.7%) (48). The Saharawi population in Sahara, Africa, had the highest CD prevalence in the world (5.6%) (49). It is well known that many people go undiagnosed due to unclear symptoms, which is also known as silent CD (50). CD is more prevalent in women than in men (60–40%). Dermatitis herpetiformis (DH) affects 10–15% of CD patients and is characterized by herpetiform clusters of pruritic, urticated papules and vesicles on the skin. Individuals suffering from CD have intestinal lining damage with the disappearance of the villi, resulting in a flattened surface and nutrient absorption limitations that cause deficiencies. The only remedy is a lifelong adherence to a strict gluten-free diet.

The role of oats in CD is still under discussion. Storage proteins in oat seeds differ from those in wheat, barley, and rye (51), and avenins, the oat prolamins, are significantly lower than in wheat and other gluten-containing cereals. Oats contain epitopes with the potential to trigger CD (52), albeit in low concentrations (53), as well as immune-reactivity (45). Some studies found differences in the immunological responses of different oat varieties (53, 54). Recent evidence suggests that oats cultivated and harvested under controlled conditions, avoiding gluten contamination, are safe for CD patients (55–57). Others, however, argue that the amount of oats consumed by people with CD should be limited for safety reasons (58). Nonetheless, gluten-free oat-based products can only be labeled gluten-free according to Commission Regulation (EC) 41/2009, if the gluten content is less than 20 ppm. Gluten contamination in the food chain is a significant challenge for the food industry, frequently leading to intake levels well above the legal limit (59–66). Further, the low fructan content in oats will potentially reduce fermentation-induced gas formation by microbiota in the colon, reducing the risk of intestinal symptoms in people with NCWS and IBS, while the high nutritional value of oats can help compensate for deficits that are often inherent in gluten-free diets. Other gluten-free flour alternatives such as amaranth, quinoa, rice, maize and potato are popular raw materials, with very low immunogenic potential and immune reactivity (45, 67). The gluten-free pseudocereal buckwheat, however, is known as a potentially highly allergenic grain (67, 68).

### Wheat allergy

Wheat allergy is an allergic reaction to proteins found in wheat. This affects 0.2–1% of the population and appears to be more common in children than in adults (69). More than 80% of children outgrow their wheat allergy by the age of 8 years, and 96% before the age of 16 years (70). Accordingly, the number of adults affected by wheat allergy (0.25%) is much lower (71). Wheat allergy can be divided into several sub-categories such as baker’s asthma, contact urticaria, and wheat-dependent exercise-induced anaphylaxis (WDEIA). The prevalence of asthma (1–10%) and rhinitis (18–29%) is high among bakers who continuously inhale flour dust. For this reason, this is referred to as baker’s asthma and bakers associated rhinitis (69). Wheat-induced chronic urticaria was estimated to affect 0.5–5% of adults (72). WDEIA is a rare severe type of allergy, induced by the combination of wheat and exercise stress, but additional triggers may also be involved (97). WDEIA has a very low prevalence in children (0.017%) (98). and there are no reliable figures for its prevalence in adults (99). Noticeably, all food allergies, including wheat allergy, are more common in women than in men (100).

### Non-celiac wheat sensitivity

NCWS is described as an unspecific immune-based reaction (sensitivity) following the consumption of wheat. There has been much discussion about the prevalence of patient-self reporting NCWS. It is assumed that the global prevalence is around 10% of the population, with high variations among different countries (82). The major reason for this is that self-reported symptoms may, to a large extent, be due to belief, which is strongly influenced by the effects of social media news as well as socio-economic factors. For example, four recently published studies from South America (Brazil, Chile, El Salvador and Paraguay) with similar criteria based on a self-administered questionnaire and more than 1,000 participants each revealed quite low prevalence of around 0.5% (101), 0.98% (102), and 1.71% (103) respectively, to moderate values of 5.2% (102). In contrast, two studies from Australia showed a prevalence of 13.9 and 14.9%, respectively (104, 105). Effects of geographic region and economic development status were confirmed by the review of (106) (Table 1), showing higher prevalence for industrial countries compared to countries with emerging economies. European studies show similar results. In England and the Netherlands, self-reported NCWS prevalence’s of 13 and 6.2% were observed (107, 108). Many of the self-reporting individuals appear non-reactive to gluten when tested in a controlled situation (109). studied in an IBS subcohort, who complained of gastrointestinal symptoms after the consumption of gluten-containing foods. 6.6% of these patients were diagnosed to suffer from CD and 0.5% from wheat allergy. The remaining people were all put on a 6-month strictly gluten-free (GF) diet, followed by a reintroduction of gluten-containing grains into their diet. Despite their self-diagnosis of being gluten-sensitive, only 6.88% of this IBS subcohort (approximately 1% of the general population) appeared to have verifiable symptoms, and 86% showed no specific reaction to gluten at all after reintroduction into the diet. Accordingly, it was concluded that self-perceived gluten-related symptoms are rarely indicative of the true presence of symptoms due to gluten consumption. Diagnosis and discrimination of NCWS and IBS are challenging due to overlapping symptoms and the absence of clear biomarkers (110–112).

**Table 1:** Overview about functional gastrointestinal disorders caused by grain components.

	Celiac disease	Wheat allergy*	Non-celiac wheat sensitivity	IBS
Prevalence
~1%	0.2–1%	~10%	7–21%
Pathogenesis	Autoimmune	IgE-induced	Unspecific, partially immune-based	Diverse and not clear (low-grade inflammation and immunological alterations)
Marker	IgA anti-EMA, IgA anti-tTG, IgG anti-DGP, IgA anti-gliadin	Specific IgE antibodies	No biomarker, IgA/IgG anti-gliadin in 50% of cases	No biomarker
Genetic preposition	More than 95% DQ2-DQ8 HLA positive	About 50% DQ2-DQ8 HLA positive	DQ2-DQ8 HLA negative	Not clear
In/uptake	Oral	Oral, respiratory & percutaneous	Oral	Oral
Source/trigger	Peptides from gliadins and glutelins	Depending on type of allergy gliadins, glutelins and/or non-gluten proteins (e.g., ATIs)	ATIs, possibly gluten and FODMAPs, predominately fructans	FODMAPs, ATIs and gluten are potential triggers
Therapy	Gluten-free diet	Wheat-free diet	Wheat-free or strong reduction of wheat in diet	Gluten-free and low FODMAP diet

*WDEIA (wheat-dependent exercise-induced anaphylaxis), contact-urticaria and baker’s asthma are summarized under wheat allergy (10, 34, 47, 69, 73–82).

### Irritable bowel syndrome

According to the World Gastroenterology Organization Global Guidelines (113), IBS is a functional bowel disorder in which abdominal pain or discomfort is associated with defecation and/or a change in bowel habit. IBS represents the most commonly diagnosed functional gastrointestinal disorder with an estimated global prevalence of about 1 in 10, ranging from 7 to 21%. A recently published systematic review and meta-analysis reported a global prevalence of 9.2% based on 53 studies from 38 countries and comprising 395,385 participants using the Rome III criteria, which are used to diagnose and classify IBS (114–117). Typical symptoms, such as bloating, abdominal discomfort, diarrhea, and constipation, overlap significantly with those of CD and NCWS (110, 118).

The recently established Rome IV criteria, classifies IBS patients into specific subgroups based on their predominant bowel habits: (1) IBS with predominant constipation (IBS-C), (2) IBS with predominant diarrhea (IBS-D), (3) IBS with mixed bowel habits (IBS-M), or (4) unspecified/unsubtyped (IBS) (115). Because IBS is not a well-defined disease, but rather a cluster of symptoms caused by multiple pathologies, accurate diagnosis is difficult, and prevalence estimates are often based on self-diagnosis. Because there is still no standardized test or biomarker for diagnosis, an examination of the detailed patient history is essential for an appropriate diagnosis. Within its complexity, it is often impossible to define exact triggers of IBS. Most patients assume that their symptoms are caused by specific foods, in particular foods rich in rapidly fermentable carbohydrates such as fruits, vegetables, pulses and grains (119). In addition, IBS and fructose malabsorption appear to overlap (120) (121). suggested that more than 50% of patients with IBS might have an atypical food allergy, despite negative skin tests and serologic analysis of immunoglobulin E. People suffering from IBS or NCWS may benefit from a significant reduction in grain-based foods containing gluten and FODMAPs (80, 81, 122).

## Components causing adverse reactions

Cereals consist mainly of carbohydrates, proteins and lipids, and are also a good source of fiber, minerals and antioxidants. While these components are the foundation of most people’s diet, wheat consumption may also induce adverse reactions in a small subset of the population. Several wheat components have been identified as potential allergens or triggers of CD and NCWS. Due to the presence of gluten proteins, even rye, barley and triticale have to be excluded from the diet. Many of these components have complex structures that are resistant to digestion and relatively stable to heat and acid exposure. Among the variety of grain components that cause wheat-related diseases, proteins are most frequently associated with wheat sensitivity. Wheat contains at least 114 allergenic proteins^1^ encoded by all three genomes (A, B and D). However, the contribution of individual components to wheat-related diseases has mostly been assessed only in *in vitro* or in animal models using isolated protein fractions, which often contain other unidentified components and/or do not reflect adulterations by processing (e.g., heat, hydrolysis by fermentation). In addition to proteins, so called FODMAPs (fermentable oligo-, di-, monosaccharides and polyols) are potential triggers of IBS and maybe even of NCWS (6, 80, 118, 123, 124). Further, a FODMAPs-rich diet is likely to exacerbate symptoms in NCWS (118).

Today, a gluten-free diet is considered the most commonly used therapy. An international threshold of 20 ppm gluten in food is generally considered to be safe for individuals suffering from CD and regulatory bodies have adopted this level for allowing food to be labeled “gluten free.” However, in severe cases of CD lower levels can result in adverse reactions. In addition, it has been shown that foods labeled to be gluten free may contain significantly more than 20 ppm due to contamination in the food chain (60, 66, 125, 126). Furthermore, gluten free-labeled foods may also contain significant amounts of FODMAPs and cause problems in patients who believe to be sensitive to gluten but in fact are sensitive to the effects of rapid carbohydrate fermentation and related intestinal discomfort (127).

### Prolamin proteins

With the exception of oats and rice, the main storage proteins of all cereal grains are prolamins. This term is based on the fact that prolamins are high in proline and glutamine. During gastrointestinal digestion, the enzymes present in the human intestine only partially digest the proline-rich sections (128), which can easily trigger adverse reactions in predisposed individuals (122). Almost all food allergens are proteins that tend to be resistant to degradation by heat, proteases, or acid hydrolysis. Thus, a large number of wheat allergens can be found among the group of prolamins (41, 129). Gluten proteins and ATIs have been identified as components involved in CD and NCWS. Gluten peptides, which remain undigested, contain repeated amino acid sequences, also referred to as CD-active epitopes, which are recognized by immune cells and induce a cascade of immune and inflammatory responses when exposed to the gut epithelium. Among them is 33-mer, an α_2_-gliadin peptide that is highly resistant to proteolysis (130) and is often described as a very potent contributor to gluten immunotoxicity (131). The number, type, and distribution of epitopes may influence the potency of the inflammatory and immune responses involved in the etiology of CD (132).

### Non-prolamin proteins

In addition to prolamins, cereals and cereal-based products contain a variety of other ingredients that may cause adverse physical reactions in sensitive people. Data from *in vitro* and animal studies suggest that non-prolamin proteins such as ATIs, serpins, lectins and others, also play or may play a role in triggering adverse gastrointestinal and extraintestinal reactions (40, 133, 134). With respect to wheat allergy, ATIs and non-specific lipid transfer proteins (nsLTPs) can sensitize susceptible atopic patients after ingestion or inhalation (37, 41, 129, 135–139). Similar to prolamins, the clinical relevance of ATIs and nsLTPs is attributed to their resistance to proteolysis and acid or heat exposure. *In vitro* studies revealed that, in addition to the well-recognized immune reaction to prolamins, CD is also associated with a humoral immune response directed against serpins (serine protease inhibitors), purinins, ATIs, globulins, farinins and lectins, also known as wheat germ agglutinin (40, 43, 44, 140). However, lectins are not heat stable and lose their adverse biological effects as a result of heat exposure, such as cooking or baking (140).

ATIs are thought to be involved in the etiology of NCWS by mediating intestinal inflammation via binding to the toll-like receptor 4 (TLR4) (45, 134). ATIs have attracted great research interest due to their suspected contribution to baker’s asthma (a respiratory form of wheat allergy), CD, and NCWS, as extensively reviewed by Geisslitz et al. (10). Although non-prolamin proteins make up a small proportion of cereal proteins, several of these proteins were suggested to be involved in adverse reactions and may contaminate protein preparations used for clinical trials, leading to erroneous conclusions about cause-effect relationships. There have been no studies to date, in which wheat-sensitive people have been tested *in vivo* for their response to individual non-prolamin components or combinations thereof.

### FODMAPs

In addition to cereal proteins, wheat contains fructans that are indigestible carbohydrates. They are classified as FODMAPs, which include oligosaccharides (including fructans and raffinose-family oligosaccharides), disaccharides (lactose), monosaccharides (fructose), and polyols (e.g., sorbitol and mannitol). Lactose is conditionally digestible in humans, as approximately 70% of human adults are lactase-non-persistent, which means lactose is a non-digestible disaccharide due to the lack of brush border β-galactosidase. Approximately 15% of the population are fructose-malabsorbers, meaning that ingested fructose is not completely absorbed in the small intestine or is absorbed only completely when glucose is also present at the same time. In these individuals, unabsorbed fructose (excess of fructose in respect to glucose) is classified as FODMAPs (141). Although fructans of grammian type, they have both β(2–1) and β(2–6) fructosyl linkages and a rather complex, branched structure, are considered to be the most prevalent FODMAPs in monocot grains (142); other FODMAPs, particularly raffinose, are present in smaller amounts and mannitol is additionally produced during sourdough fermentation. Fermentable carbohydrates are beneficial to most people’s intestinal health because they are converted to health-beneficial short chain fatty acids, while also improving peristalsis and defecation. However, whether FODMAPs ingestion is beneficial or harmful is dose and individual dependent. A quantity of >15 g of FODMAPs per day results in quantitative effects on osmosis induced liquid accumulation in the terminal small intestine and on fermentation in the terminal cecum and colon, which may cause bloating, flatulence and abdominal discomfort in susceptible people. Patients suffering from IBS appear to develop central sensitization, manifesting as pain hypersensitivity (143), a reason why they may experience symptoms at lower doses of FODMAPs compared to non-sensitized individuals. It is estimated that cereal-based products may account for up to 70% of the daily fructan intake in the United States (144), with intakes varying across populations depending on the amount of bread and other grain-based foods consumed (145). Depending on other foods consumed that contribute to the FODMAPs intake (such as fruits, vegetables, pulses, artificially sweetened soft drinks, and apple juice), the contribution of grain fructans may be minimal to significant (141, 146–148). Low-fructan meals may help to reduce intestinal gas formation and alleviate symptoms in IBS patients (149–157).

## Sourdough and microbial communities

### Sourdough fermentation

In baking applications, sourdough is used for different technological reasons. Only few countries, mainly in the European Union, established a legal definition of sourdough and the approach to the definition of sourdough varies among different countries. France, Spain and the Netherlands define sourdough by specifying a maximum level of baker’s yeast addition and a minimum level of acidity as defined by either pH, total titratable activity or acetic acid concentration. Austria, the Czech Republic and Germany define sourdough as a dough with metabolically active lactic acid bacteria (158). An overview of the different use of sourdough in baking applications is presented in Table 2. Owing to the different use and definition of sourdough in baking application, the term “sourdough bread” includes fermentations that use diverse microbes and subject a different proportion of the flour to fermentation. With type I sourdoughs, sourdoughs that are used as sole leavening agent, 20–30% of the flour are fermented for more than 6 h and the time for proofing generally exceeds 2 h. With type II sourdoughs, sourdoughs that are used for dough acidification and/or for improved bread quality, usually 10–20% of the flour is fermented in the sourdough fermentation but sourdough microbes have a lower metabolic activity in the bread dough when compared to type I sourdoughs. Type III sourdoughs, dried sourdoughs without viable and metabolically active microbes, are added at a level of 3–5%. Here, only a small portion of the flour is fermented with lactic acid bacteria and metabolic activity in the bread dough is attributable only to baker’s yeast. Freeze dried starter cultures are commercially available but do not show appreciable metabolic activity in the bread dough unless they are propagated (159).

**Table 2:** Use of sourdough in baking and microorganisms in sourdoughs.

	Type 0 sourdoughs (levain, poolish or sponge dough)	Type I sourdoughs	Type I sourdoughs	Type II sourdoughs	Dried sourdoughs	Starter cultures
Technological aim of fermentation	Improved flavor, reduced addition of baker’s yeast	Leavening agent in bakeries	Leavening agent in households	Acidification and improved flavor in (industrial) baking	Spray-dried or drum-dried sourdough without active microbes	Acidification or flavor
Inoculum	Baker’s yeast	Back-slopped	Back-slopped	Back-slopped	Back-slopped or defined strains	Freeze dried defined strains
# of fermentation cycles per day	n/a	2–4	0.1–0.3 (refrigerated storage)	0.2–1	n/a	n/a
proofing time addition baker’s yeast	1–2 h 0.5–2%	2–3 h 0–0.5%	2–3 h 0–0.5%	1–2 h 0.5–4%	1–2 h 1–4%	Variable
% of flour in sourdough	10 to >20%	20–30%	20–30%	10–20%	1–5%	Variable
Key bacterium	*Lactiplantibacillus plantarum*	*Fructilactobacillus sanfranciscensis*	*Fl. sanfranciscensis*	*Limosilactobacillus pontis Lactobacillus amylophilus*	Depending on inoculum	Depending on inoculum
Other bacteria	*Latilactobacillus sakei Pediococcus* sp.	*L. plantarum Levilactobacillus* sp. *Companilactobacillus* sp. *Leuconostoc mesenteroides*	*L. plantarum Levilactobacillus* sp. Compani-other lactobacilli *Pediococcus* sp.	*L. panis*, *L. reuteri*, *L. fermentum*; *L. crispatus*, *L. acidophilus*		
Key yeast	*Saccharomyces cerevisiae*	*Kazachstania humilis*	*S. cerevisiae*	none	None	None
Other yeasts	*-*	*S. cerevisiae K. exigua*	*K. humilis*	*S. cerevisiae*		

### Microorganisms in sourdough

Currently, reliable data on the composition of more than 1,000 sourdoughs that have been used in bakeries or at the household level are available (160). Collectively, these data provide a comprehensive overview on microorganisms in sourdough. The geographical location has no impact on microbial communities in sourdoughs. The type of flour also has remarkably little influence on microbial communities in sourdough as long as whole flours or extracted flours from wheat and rye are used. In flour from other grains, the lack of maltogenic amylases and/or the presence of phenolic acids with antimicrobial activity selects against some sourdough microbes, particularly *Fructilactobacillus sanfranciscensis* (161, 162).

Overall, more than 100 species of lactic acid bacteria, by far predominantly species in the *Lactobacillaceae* (89) and several dozen yeast species have been isolated from sourdoughs. The genera *Fructilactobacillus*, *Lactobacillus*, *Limosilactobacillus* and *Lactiplantibacillus* dominate most sourdoughs. *Companilactobacillus*, *Lacticaseibacillus*, *Latilactobacillus*, *Pediococcus*, *Levilactobacillus*, *Lentilactobacillus*, *Furfurilactobacillus*, *Weissella* and *Leuconostoc* are frequently isolated. Species of other genera are not found in sourdoughs or were isolated in laboratory sourdoughs only. Generally, sourdough microbes include two to six major representative species, which represent stable associations of heterofermentative and homofermentative *Lactobacillaceae* with the former typically more abundant. Despite the apparent diversity of sourdough microbiota, several types of sourdough have a globally uniform microbial composition that converges at the genus or even species level. The composition of the microbial consortia depends on the fermentation conditions, which, in turn, are dictated by the technological aim of the fermentation (Table 2).

To use sourdough as sole leavening agent, sourdough microbes are maintained at their peak metabolic activity by frequent back-slopping. In bakeries, the process of frequent back-slopping is interrupted only on 1 day per week (163, 164). This fermentation scheme selects for those organisms that grow fastest in wheat or rye doughs and microbial consortia in type I sourdoughs are globally uniform at the species level. A vast majority of type I sourdoughs includes *Fructilactobacillus sanfranciscensis* as main bacterial representative and *Kazachstania humilis* as the main representative of yeasts. Other bacterial species that are frequently isolated include *Lactiplantibacillus plantarum*, *Levilactobacillus* species, *Companilactobacillus* species, *Weissella* species and *Leuconostoc* species (160). Extensive baking by farmer-bakers, i.e., four or fewer baking days per week, may result in selection of *Kazachstania* species other than *K. humilis* (165). Amateur bakers at the household level include more extensive interruptions of the continuous back-slopping process and use only 5–10 back-slopping cycles per month. The microbial consortia in these sourdoughs depends on the diverse fermentation conditions of the individual bakers. Lactobacilli other than *Fl. sanfranciscensis* are likely to predominate and *S. cerevisiae* is the most prevalent yeast (Table 2) (164). Acetic acid bacteria were also shown to occur in sourdough (166). As strict aerobes, their growth is restricted to the surface and their contribution decreases with increasing scale of the fermentation, corresponding to a smaller surface to volume ratio.

Most installations for industrial type II sourdough fermentations are found in Central, Northern and Eastern Europe where rye bread is common. In rye baking, dough acidification is essential to inhibit flour amylases, and to solubilize arabinoxylans, while leavening is achieved by addition of baker’s yeast (159). High levels of acidity are often achieved by fermentation at more than 30° C and long fermentation times; these conditions select for *Limosilactobacillus* and *Lactobacillus* species with *Lm. panis* and *L. amylovorus* being among the most frequent species (Table 2) (163, 167). Yeasts may or may not be present and, if present, *S. cerevisiae* is the most frequent organism (Table 2) (163, 168–170).

Freeze dried cultures are available as starter cultures (Table 2) but exhibit sufficient metabolic activity only after back-slopping and are therefore not widely used (Table 2). Dried sourdoughs are produced by ingredient suppliers for use in bakeries, which are either obtained by spray-or drum-drying with supersaturated steam (up to 130° C) (158, 171). Different producers of dried sourdoughs use either back-slopped sourdoughs that resemble type II sourdoughs, or inoculate the sourdoughs with defined strains. Dried sourdoughs are used at an inclusion level of 3–5% based on flour and, because sourdough microbes are killed during drying, they exhibit no metabolic activity in the bread dough. Nevertheless, aroma and shelf-life of bread are improved by key flavor compounds and lactic acid from dried sourdoughs (172).

### Reduction of potentially harmful components by sourdough fermentation

The allergenic, immunogenic and inflammatory potential of wheat components is primarily due to their structure. Most processing procedures, including sourdough fermentation, have the potential to alter the quantity and structure of wheat components, which may affect the way these components are presented to the intestinal epithelium and immune system. Food processing affects wheat allergenicity (173); however, preclinical validations and human clinical studies are currently lacking. Although there is no validated strategy for producing cereal-based sourdough-fermented foods with lower levels of allergens and triggers of CD and NCWS, experimental sourdoughs have shown reductions that potentially may reduce adverse reactions (10, 13, 174–176).

### Protease activity and changes in protein digestion

An extensive review of proteolytic aspects of grains, flours and fermentation media was provided by Gänzle et al. (175). In short, grains contain endogenous proteases (aspartic proteinases, carboxypeptidases, and cysteine proteinases) which are active at lower pH values and are important during the germination of the grain (seed) for the formation of amino acids required for the growth and development of the crop (177, 178). These enzymes are activated by the acidic conditions of dough making. Lactobacilli in sourdough express intracellular protease activity (175), which induces significant degradation of peptides, including toxic gluten epitopes, resulting in an almost linear increase in amino acids with fermentation time (179–184). This increase in free amino acids in sourdough contributes to the improved flavor and taste effects of baking sourdough bread (183). Only type I and type II exhibit significant proteolysis; in other types, either the dough pH never falls below 4.5, i.e., the pH below which cereal proteases are activated, or the proportion of flour fermented with sourdough is too low to affect the overall composition of the bread.

**Figure 3:**
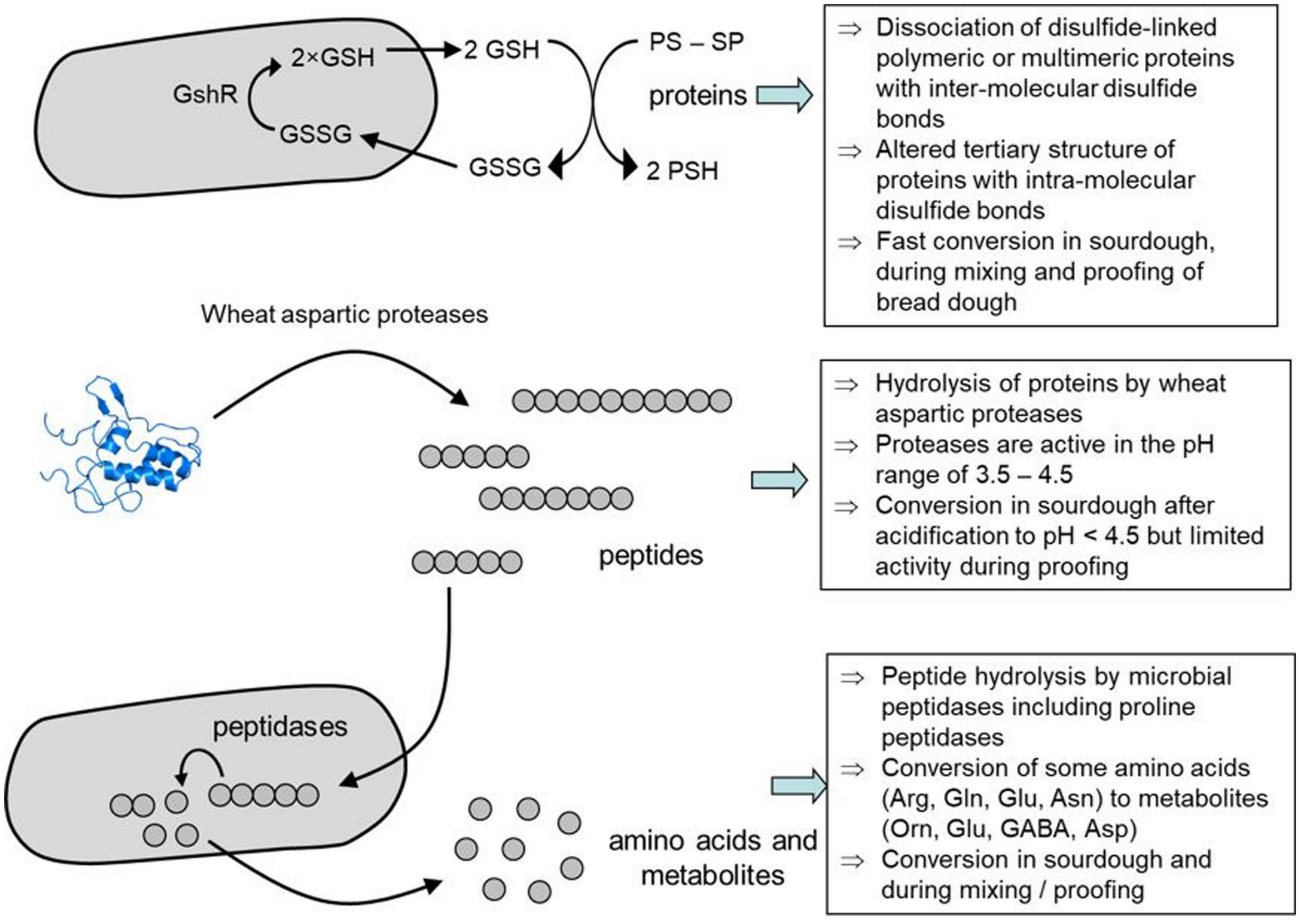
Mechanism of wheat protein degradation by sourdough fermentation. Modified from Gänzle et al. (175) with illustrations from https://www.ebi.ac.uk/pdbe/entry/pdb/1bip/protein/1.

In addition to proteolytic activity, reducing power generated by sourdough microbes impacts protein stability and proteolytic degradation (Figure 3). Heterofermentative lactobacilli accumulate reduced glutathione, or produce other low-molecular weight thiols that interact with glutathione to reduce intra- and intermolecular disulfide bonds (185). Thus, gluten proteins depolymerize (175) and impacts bread volume (186, 187). The glutathione reductase of *Fl. sanfranciscensis* has been found to enhance the degradation of wheat germ agglutinin and allergenic ovotransferrin in sourdoughs (188, 189). The reduction of disulfide bonds to thiol groups decreases the resistance to proteases and potentially alleviates adverse events such as inflammation, immune and allergic responses that are dependent on proteins with an intact tertiary structure, either for activity or for passage through the intestinal tract. The reduction of intramolecular thiol groups during the breadmaking process, however, has been documented only for type I sourdoughs.

Following food consumption, protein digestion takes place via the combined action of stomach acid and gastrointestinal enzymes (pepsin, trypsin, chymotrypsin), in combination with brush border peptidases. Post-consumption, specific food-derived proteins and peptides may resist degradation due to the lack of specific gastrointestinal enzymes required for their complete degradation. Grain proteins that are particularly high in disulfide bonds and/or proline, such as gluten proteins, are examples. The human gastrointestinal tract lacks the proline-specific peptidases required to cut the post-proline bonds in the gluten peptide chains (190), resulting in incomplete digestion and leaving proline-rich gluten fragments intact. These fragments induce immune responses and inflammation, causing CD. They are often referred to as toxic gluten peptides (epitopes) that can be recognized by the immune system. A reduction of gluten epitope levels in food by proline-specific peptidases during food processing, may help reduce adverse reactions. Many lactobacilli as well as fungi, including *Aspergillus oryzae*, express proline-specific peptidases that cleave peptide bonds adjacent to proline (191–195). However, sourdough products available on the market are generally not processed in this way, and there is no data to support the claim that sourdough breads reduce the risks of developing celiac disease.

### Conversion of FODMAPs

Sourdough fermentation alone or in combination with yeast leavening is an effective approach to reduce FODMAP levels in wheat bread. The major FODMAPs in wheat flour are fructo-oligosaccharides with a degree of polymerization of 3 to 10, which are present at 1–3% and 3–5% in wheat and rye, respectively, and raffinose, which is present at 0.2–0.7% (95, 196, 197). An overview of enzymes that degrade or generate FODMAPs in breadmaking is presented in Table 3. Studies revealed that lactobacilli or yeasts with extracellular fructanases show the most extensive degradation, with a reduction of the fructan content in wheat flour by up to 95% during the breadmaking process. A sourdough that includes lactobacilli expressing the exceptional extracellular fructanase FruA, however, is maintained only by one bakery as reported by (85). Yeasts with extracellular fructanase activity have been used experimentally but not in commercial bread making (84). Fructan hydrolysis by extracellular fructanases generates fructose, which is included in the FODMAP definition when present in excess of glucose, which is not the case in bread after prolonged fermentation and baking (11, 198). Baker’s yeast converts up to 2.5% fructose to ethanol and carbon dioxide in the bread making process (85, 199). Yeast invertase is known to hydrolyze fructo-oligosaccharides but the specificity on oligosaccharides other than sucrose is highly strain-specific (11, 87) and it remains unknown whether yeasts that were domesticated in sourdough fermentations efficiently degrade fructans (200). Intracellular fructanase activity is present in many homofermentative lactobacilli but intracellular enzymes are active on oligosaccharides only after cell lysis (90). Sucrose phosphorylase, which is present in most lactobacilli and the main route of sucrose conversion in heterofermentative lactobacilli, is not active with fructo-oligosaccharides other than sucrose (91, 201). Heterofermentative lactobacilli convert fructose to mannitol but the mannitol concentrations in sourdough bread are generally below 0.3 g/kg and may be further reduced by homofermentative lactobacilli that utilize mannitol as carbon source (95). Raffinose is metabolized by levansucrase or sucrose-phosphorylase activity of lactobacilli in conjunction with α-galactosidase (92). Raffinose levels in wheat flour are reduced by more than 50% independent of the fermentation organisms (85, 202).

**Table 3:** Enzymes of sourdough microorganisms with activity on FODMAPs in wheat and rye.

Enzyme	Organism	Extend of FODMAP conversion
Extracellular fructanase	Strain specific in *L. crispatus*, *L. amylovorus*, and *L. paracasei*; *Kluyveromyces lactis*	Rapid hydrolysis of all fructans; the enzyme in *L. paracasei* is repressed by glucose and unlikely to be active in sourdough^1,2,3,4^
Extracellular invertase	*S. cerevisiae*, other yeasts including *K. humilis*	Activity and substrate specificity strain-dependent in *S. cerevisiae*^5,6^ and unknown in *K. humilis*
Intracellular fructanase	Most homofermentative lactobacilli	Active only after cell lysis or on di-, tri-and tetrasaccharides that are transported to the cytoplasm^7,8^
Intracellular sucrose phosphorylase	Most lactobacilli	Active on sucrose only^9^
Levansucrase or sucrose phosphorylase and α-galactosidase	Most sourdough lactobacilli but not *Fl. sanfranciscensis*.	Hydrolysis of raffinose, stachyose and verbascose^10^
Mannitol dehydrogenase	Most heterofermentative lactobacilli but not *Weissella* spp.	Highly specific conversion of fructose to mannitol^11,12^
Mannitol-1-phosphate dehydrogenase	Many homofermentative lactobacilli, exceptional in heterofermentative lactobacilli	Utilization of mannitol as carbon source^12,13^

^1^Loponen et al. (83), ^2^Struyf et al. (84), ^3^Li et al. (85), ^4^Goh et al. (86), ^5^Laurent et al. (87), ^6^Nilsson et al. (88), ^7^Zheng et al. (89), ^8^Ganzle and Follador (90), ^9^Teixeira et al. (91), ^10^Teixeira et al. (92), ^11^Gänzle (93), ^12^Qiao (94), ^13^Loponen and Ganzle (95).

FODMAPs reduction of less than 50 to more than 95% have been reported. In yeast leavened bread, the extent of fructan degradation is mainly dependent on the fermentation time and the invertase activity of the baker’s yeast. Extended proofing times were shown to suffice to degrade a substantial part of wheat fructans. Sourdough bread is more commonly fermented with extended proofing times and the strain-specific capacity of sourdough lactobacilli for fructan hydrolysis additionally degrades FODMAPs (84, 85, 87, 196, 202, 203).

In conclusion, reducing FODMAPs through sourdough fermentation can help alleviate gastrointestinal distress in sensitive individuals. FODMAP levels in processed cereal products can be compared to cut-off values established by Varney et al. (141), which forms the foundation for classifying foods as being “low FODMAPs products.” Laatikainen et al. (6) investigated the effects of sourdough bread on IBS symptoms. Flatulence, abdominal pain, cramps, and stomach rumbling were all reduced after consuming low-FODMAPs rye sourdough bread. A subsequent study confirmed the degradation of ATIs and FODMAPs during sourdough fermentation, but sourdough bread was not better tolerated than straight dough bread (7). Furthermore, comparison of a regular rye sourdough with a specific sourdough containing unique lactobacilli that efficiently metabolize fructans showed no significant improvement of symptoms (124). Thus, the clinical relevance of sourdough bread for FODMAP-sensitive individuals remains unclear and the effects may be more dependent on the composition of the overall diet.

## Reduction of anti-nutritive compounds and contaminants

### Degradation of phytate, wheat germ agglutinin and amylase trypsin inhibitors

In many plant tissues, including grains, phytate or phytic acid (inositol-6-phosphate), stores phosphorus and minerals. High levels of phytate in cereals reduce mineral bioavailability in the small intestine because phytate chelates minerals such as Zn^2+^, Fe^2+^, Mg^2+^, Ca^2+^, Mn^2+^ and Cu^2+^. Furthermore, by binding amino acids, peptides, and enzymes, phytate may also influence amino acid and carbohydrate metabolism (204).

During sourdough fermentation, the dough is acidified to a pH less than 5.0, which solubilizes phytate salts of Fe^2+^, Mg^2+^ and Ca^2+^ while maintaining the activity of cereal phytases close to their optima (205–208). Improved bioacessibility after sourdough fermentation was confirmed by *in vitro* experiments (209). Consumption of sourdough bread increased mineral absorption in rats when compared to a diet based on yeast-fermented bread (210). The exact impact of sourdough on mineral absorption in humans can differ depending on a variety of factors, including the type of grain used, the fermentation time, and individual differences in gut physiology. However, low mineral intakes are common both in non-industrialized as well as industrialized countries; these individuals may benefit from improved bioavailability, as low Fe intake and/or bioavailability is a major risk factor for developing anemia (211, 212).

Wheat germ agglutinin, as present in raw cereal materials, is a lectin with the potential to reduce nutrient absorption and cause digestive problems (189, 213). Sourdough fermentation was reported to reduce WGA levels in dough, with the extent of degradation determined by thiol metabolism and redox potential rather than proteolytic activity (189). WGA reductions in the dough may also reduce adverse effects of lectin exposure in the intestine. Although this may be the case only for unprocessed flour or cereals, experimental studies have shown that heat exposure of more than 90° C, such as taking place during baking inactivates WGA leading to loss of bioactivity, as reviewed by (140).

ATIs are relatively heat and acid stable as well as digestion resistant. As a result, they remain largely intact during food processing and during gastrointestinal transit (10). ATIs have the ability to inhibit digestive enzymes as well as induce innate immune responses and inflammation, which is why they are suspected to play a significant role in the etiology of CD and NCWS (10, 214). Several studies documented the fate of ATIs during sourdough fermentation. However, these studies have only investigated the degradation resulting from fermentation in dough samples and not in the baked bread. Additionally, extraction protocols that do not account for denatured or aggregated proteins in bread were frequently used (14, 15, 175, 189) or model systems with protein fractions were examined (12). Therefore, data of effects of the consumption of ATIs by humans, as present in heat-processed cereal foods, analyzed appropriately, are required for drawing valid conclusions (215).

### Reduction of acrylamide and mycotoxin levels

According to Council Regulation (EEC) No 315/93 acrylamide belongs to the group of contaminants and is a carcinogenic chemical hazard in foods formed during heat induced Maillard reaction (216). The concentration of acrylamide in baked foods such as bread is mainly affected by the presence of precursors such as reducing sugars and asparagine, as well as their ratio, which is mainly determined by flour quality such as type and degree of milling, and by fermentation conditions (217). Furthermore, thermal processing conditions (temperature, and time) and the water content influence acrylamide formation (218). Thiol groups reduced acrylamide formation during baking (219). A lower pH also resulted in lower acrylamide levels after addition of low or moderate amounts of dried sourdough for bread making (220).

Lower concentrations of acrylamide were observed in wholegrain sourdough breads compared to bread fermented with yeast only. The range of acrylamide contents in sourdough bread indicated a strain specific behavior. Significant relationships were also detected between pH, TTA and lactic acid, whereas no correlation was found between the amount of reducing sugars and amino acids (221). Other studies also reported a decrease of about 25–59% and strain specific effects. *Pediococcus pentosaceus* had the highest potential to reduce acrylamide levels (222). Other studies found lower acrylamide levels in sourdough fermentation as well, but observed a significant correlation with the amount of reducing sugars (223, 224). Contrary to the previously mentioned publications, Fredriksson et al. (217) found that sourdough fermentation did not diminish free asparagine and had an adverse impact of its utilization by yeast, which results in increased acrylamide levels. However, the fermentation reduced the acrylamide content significantly, which is commonly much longer in the case of sourdough fermentation. In short, conclusive evidence on mechanisms or metabolic activities of sourdough microbes that reduce acrylamide formation during baking remains elusive but, despite the increase of the concentration of asparagine during fermentation, sourdough fermentation decreases rather than increases acrylamide formation in bread production (217, 218, 221, 222). A reduced pH and an increased concentration of thiols may be main factors for the reduction of acrylamide levels but the impact on health and the cause of its reduction remains questionable.

The usage of raw materials and flours contaminated with fungal toxins is not possible from a legal point of view, irrespective of the reduction of mycotoxin concentrations by fermentation. COMMISION REGULATION (EC) No 1881/2006 established maximum levels for main mycotoxins in foodstuffs, including cereals and bakery products, which prohibits their usage even if the concentration of mycotoxins is strongly reduced by processing. Nevertheless, a few research groups examined potential mycotoxin degradation by sourdough fermentation, which was highlighted in two recently published reviews (225, 226). Results indicated degradation of mycotoxins in different intensities by sourdough fermentation, depending on applied microbiota and conditions. Although shown results of several studies revealed a high potential of sourdough fermentation for limiting mycotoxins exposure in bread consumption, the role of LAB might not be the predominant reason for lower mycotoxin levels. A study applying yeast fermentation only for bread making already showed a significant reduction in three fungal toxins spiked in wheat flour (227). Furthermore, the baking process and the induced heat impact had a strong effect on the mycotoxin contents, as mycotoxins were diminished in a higher extent in the crust than in the crumb of breads (228).

However, data about degradation products and its possible health hazards are missing. Adsorption mechanism of cells walls may be responsible for lower mycotoxins levels. Thus, release of adsorbed toxins during human digestion cannot be excluded. Detoxification of mycotoxins other than patulin by sourdough fermentation is not supported by reliable studies on metabolic pathways or enzymatic activities of sourdough microbes with activity on mycotoxins, or by *in vivo* studies, and is therefore unlikely to be relevant (225–227, 229–234). Milling strategies seem to be more suitable for mitigating mycotoxin exposure as they could be applied for flour production from a legal point of view (235).

## Sourdough fermentation as a tool to obtain nutritional and health benefits

### Bioactive peptides and amino acid derivatives

Long-term fermentation with a reduction of the pH to less than 4.5 results in a significant degradation of proteins with a corresponding increase of amino acids and peptides. If sourdough fermentation generating reducing power is combined with addition of fungal proteases, a virtually complete degradation of proteins to amino acids and peptides can be achieved (192, 236). Some of the peptides that are released from wheat proteins during sourdough fermentation and detected in sourdough or sourdough bread were proposed to have biological activity (236–242).

**Figure 4:**
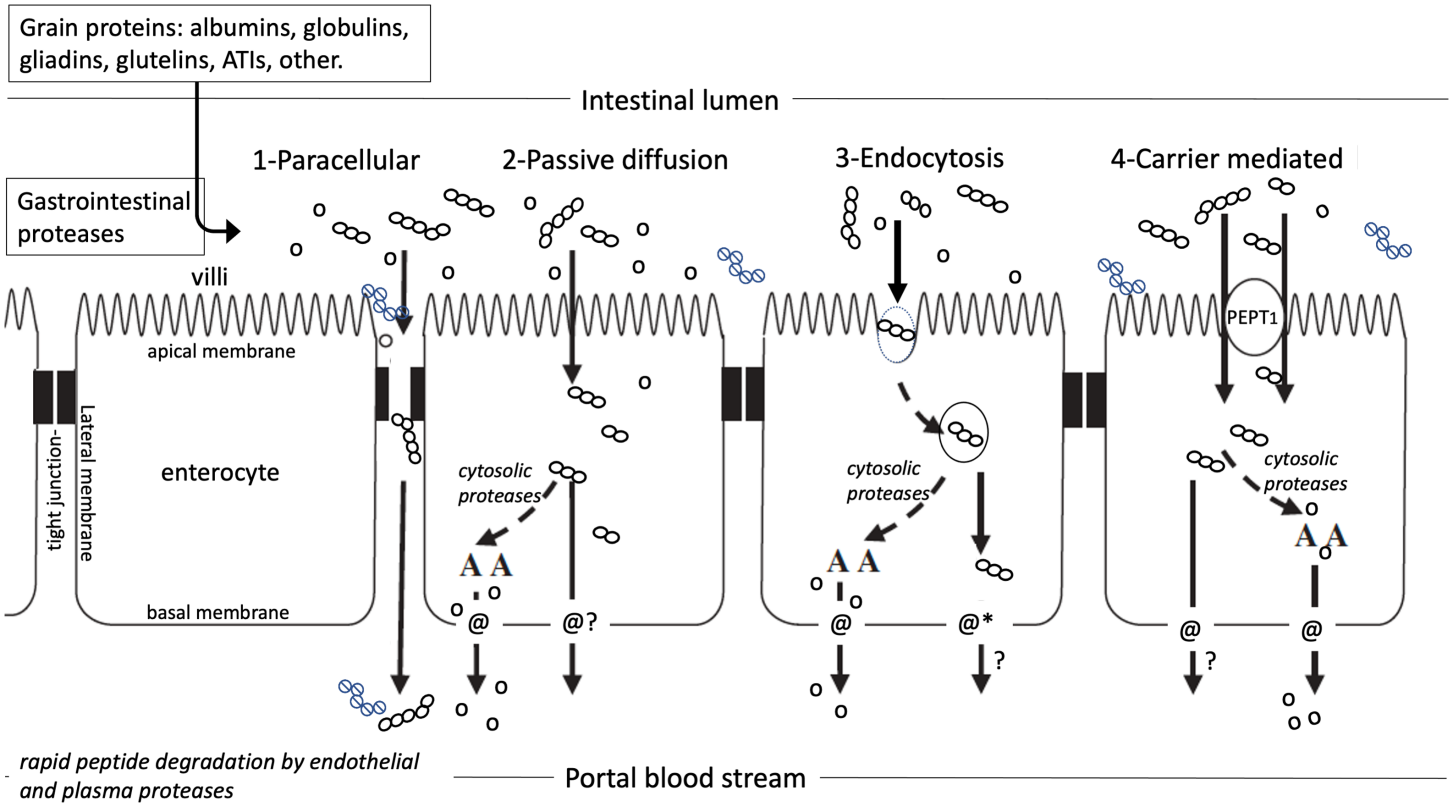
Potential pathways of amino acid and peptide absorption in the small intestine. (1) paracellular through widened tight junctions, (2) passive diffusion through the enterocyte, (3) endocytosis, followed by carrier transport or suggested peptide cargo*-permeability, (4) Transport carrier mediated passage *It is unlikely that peptides will passively diffuse across the cell membrane, but altering their physical properties (such as conformational flexibility and polarity), has been proposed to improve their permeability, also referred to as peptide cargo (243). Open dots represent amino acids, open dot-chains represent digestible peptides, closed dot-chains represent proteolysis resistant gluten peptides. Modified from Brouns and Shewry (202).

In order to exert systemic biological effects, intact food peptides must be absorbed in sufficient quantities and remain intact in blood or biofluids for sufficient time to have measurable biological activity (Figure 4). Bioactive gluten peptides were indeed shown to pass the disordered intestinal epithelium in CD patients by paracellular transport into blood after consumption of wheat protein (244). Untreated CD patients, however, have an inflamed, damaged intestinal barrier, allowing even macromolecules including intact proteins, peptides and bacteria to pass paracellularly from the intestinal lumen into blood (245–248). The presence of large peptides in blood under such specific pathophysiological circumstances does not prove that these are also absorbed under normal physiological conditions, during which most dietary peptides are rapidly hydrolysed by pancreatic enzymes and brush border peptidases. Few tri-and tetrapeptides that are rich in proline and thus resist degradation by brush border peptidases have been shown to translocate to the blood plasma; however, their concentration in plasma is less than 0.1% of the active concentration and the half-life of different peptides was determined as less than 15 min (29, 30, 249–251).

Despite the fact that dietary bioactive peptides do not pass into the bloodstream in biologically relevant concentrations, health benefits of few groups of bioactive peptides were consistently demonstrated in clinical trials. The ACE inhibitory peptides IPP and VPP reduced the systolic blood pressure in moderately hypertensive patients (252–255). Likewise, milk protein hydrolysates reduced the postprandial glucose response both when administered as a single dose, or over a period of several weeks (256–258). In both cases, the clinical outcomes likely relate to luminal or local (gastro-intestinal) effects rather than translocation of peptides to the blood. The biological effect of ACE inhibitory peptides was hypothesized to interact with the intestinal renin–angiotensin system, which regulates the passage of Na and fluid across the gut wall (30). A reduced postprandial glucose response may also be attributable to luminal effects, either to the inhibition of starch digestive enzymes by dietary peptides (259), or to interaction of dietary peptides with intestinal receptors that modulate the endocrine response and insulin secretion (260, 261).

Observations from animal and *in vitro* laboratory research, have led to the suggestion that the presence of gluten exorphins, peptides with 4–8 amino acids that have opioid activity, stimulates weight gain and decrease resting energy expenditure, resulting in weight gain, however, a critical assessment found no reliable evidence in support of this claim (262).

In summary, clinical evidence for health beneficial properties of bioactive peptides in sourdough bread is not available. When using a fermentation protocol that was optimized for proteolysis by including rye malt, fungal proteases and extended fermentation times, the angiotensin-converting enzyme inhibitors IPP and VPP were shown to be present in sourdough bread and sourdough steamed bread in clinically relevant concentrations (242). Whether their presence elicits a relevant physiological response, either alone or in conjunction with γ-aminobutyrate (GABA) or γ-glutamyl peptides, remains to be documented in future studies (192, 236, 263).

### Effects of sourdough and yeast fermentation on starch digestion and blood glucose response

**Figure 5:**
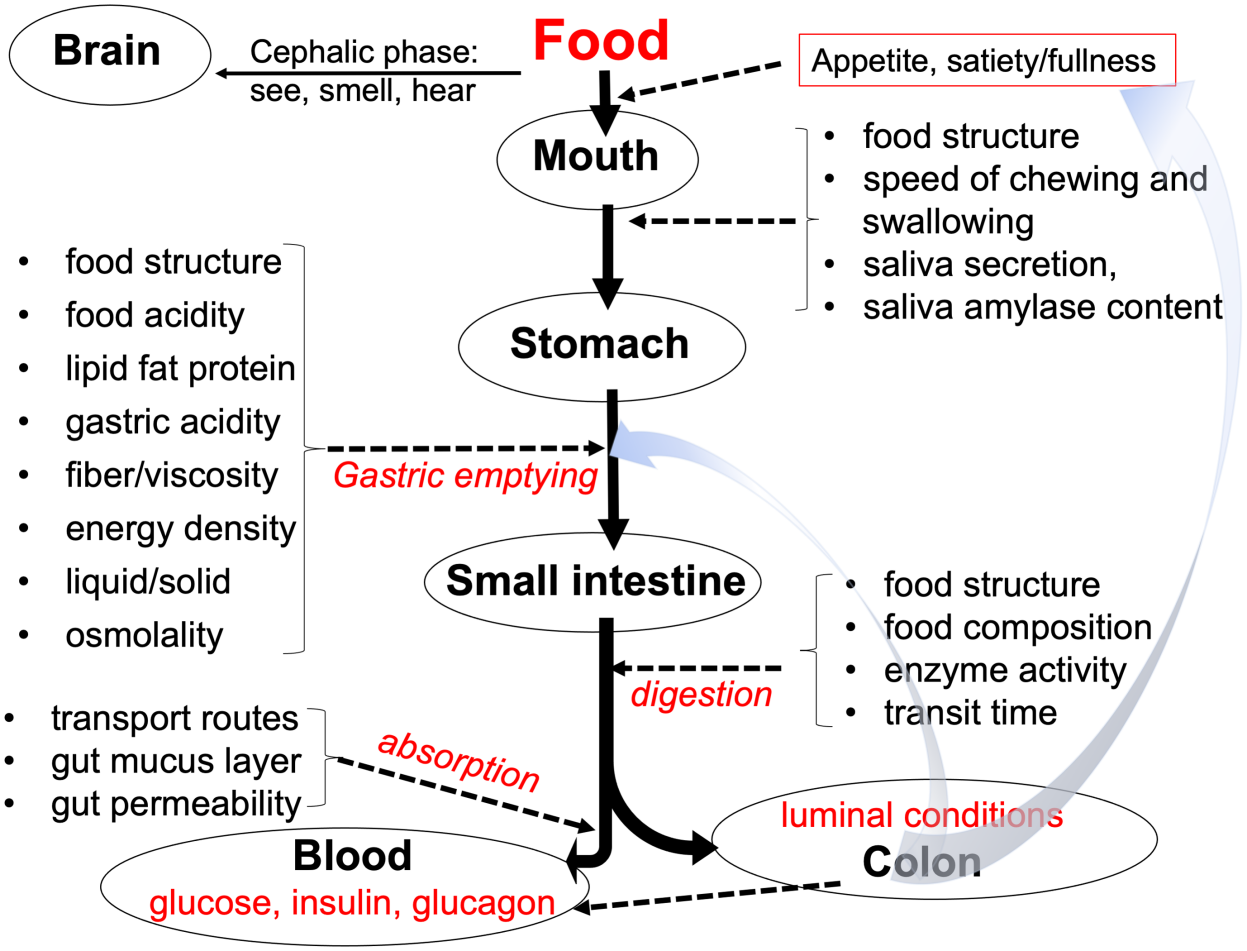
Factors that play a role in gastrointestinal meal transit, digestion, and absorption of nutrients. Adapted from Brouns (267).

The effects of an undesired high and long elevated blood glucose drive a wide range of health-related factors (264, 265). GI is a measure of the potential of carbohydrates to raise the level of blood glucose and is determined by ingesting a food portion containing a calculated amount of 50 g of available carbohydrates followed by measuring the area under the blood glucose-response-curve above baseline (Table 4) (266). Influencing factors include the overall macronutrient composition, processing, matrix characteristics, and the content of active enzyme inhibitors (as illustrated in Figure 5).

**Table 4:** Glycemic index value of bread and some other carbohydrate sources, as tested vs. glucose as reference-control.

Source/food	Glycemic index (GI)
Glucose	100
Bread: French baguette, wheat	95
Bread: Sourdough, whole grain, rye	53
Bread: White wheat flour, mean of 16 studies	75
Bread: Whole grain rye, mean of 4 studies	58
Bread: Whole grain wheat, mean of 10 studies	74
Pasta Macaroni, white boiled, mean of 3 studies	50
Pasta Spaghetti, white boiled, mean of 8 studies	41
Potato boiled, mean of 7 studies	53
Rice, white, mean of 8 studies	53
Sweet potato	61

Atkinson et al. (96) and University of Sidney (accessed 2020) online searchable data GI, International Tables of Glycemic Index and Glycemic Load Values.

Human GI testing must follow strict ethical guidelines, is time-consuming, and costly. Hence, *in vitro* digestion has been used to predict responses in humans. Data obtained from *in vitro* digestion of bread predicted *in vivo* GI values relatively well (268), which is in consistence with findings of others (269). However, when looking at absolute figures of the digestion rate (glucose release) or glycemia, there may be differences. Vangsøe et al. (270) showed that the *in vitro* digestion method ranked the tested diets in a similar relative manner as *in vivo*. The rate of glucose release *in vitro*, however, was much faster than the portal glucose appearance *in vivo*. In swine, the data on the *in vitro* release of glucose from starch correlated with portal glucose appearance rates only after correction for gastric emptying rate (271). Hence, the effects of gastric emptying have to be considered. Therefore, data from *in vitro* digestion and predicted GI should be considered as only indicative of what may be expected *in vivo* in humans. Different *in vitro* studies on different types of bread raise questions about the validity of benefit claims communicated in marketing. In fact, Korem et al. (5) found no significant effect of glycemic response after consumption of sourdough fermentation bread vs. yeast fermented bread in a crossover trial. While bread consumption did affect clinical parameters, the glycemic response to the different bread types varied greatly among the participants.

### Effects of dietary fiber in kernels and flour on glycemic effects

The content and type of dietary fiber in the food matrix are known to influence the gastrointestinal transit, intestinal viscosity, and the rate and magnitude of macronutrient digestion and absorption. With respect to starch, this may result in a reduction of the blood glucose response (272). Hence, it is often suggested that wholemeal based breads result in a lower GI compared to white flour-based breads, low in fiber, which are based on observations that dietary fiber enriched sourdough-bread resulted in lower GI (< 55) values (273–276).

However, fibers of different cereals differ significantly in composition. Oat fiber consist of about 30% soluble and 70% insoluble fiber types, mainly cellulose, arabinoxylan, β-glucan, xyloglucan and fructan. The soluble fibers express viscous properties that are beneficial for slowing the glucose absorption rate in the intestine. This part of the fiber is also well fermentable and impacts on microbial production of short chain fatty acids, which are known to modulate metabolism and insulin sensitivity, in favor of overweight and diabetes risks reduction (277–280). A systematic review and meta-analysis addressing the effects of dietary fiber and whole grains in diabetes management concluded that higher-fiber diets are an important component of diabetes management (281). It appeared, however, that these effects were independent of the type of dietary fiber. In addition, the impact of fiber on health outcomes depends on the presence of other phytochemicals that are present in whole grains, particularly phenolic compounds (282). Because of these beneficial effects, adding fiber to the dough has become an important target of study. Owing to the beneficial impact of sourdough fermentation on the technological functionality of fiber-rich ingredients, whole grain sourdough bread is an optimal format for further studies.

### The fate of resistant starch in sourdough fermented dough and bread

As a result of food processing, a fraction of digestible starch may be transformed into resistant starch, causing a decrease in the glycemic potential. The resistant starch fraction comes on top of a fraction of starch in bread that remains undigested during transit in the gastrointestinal tract (usually 3–5% of total starch) and passes on to the colon. These fractions will be completely fermented and will serve as an important substrate for the colonic formation of butyrate, which has been linked to gastrointestinal health and disease reduction (283, 284).

Available data show mixed outcomes in terms of the dough stage. De Angelis et al. (275) reported a fermentation-induced increase in resistant starch, whereas Liljeberg et al. (285) found no difference. Furthermore, Hefni et al. (268) observed no change in resistant starch content during dough formation but an approximately 3-fold increase during the subsequent step from proofed loaf to bread. A recent study used X-ray diffraction and FTIR to determine the crystallinity of starch from white breads fermented with yeast only or sourdough. Results indicated higher total crystallinity in sourdough bread due to the formation of resistant starch. To eliminate confounding factors, breads were baked identically and the temperature was set for storage experiments (286). Da Ros et al. (287) compared straight dough and sourdough bread baked under the same conditions and reported a higher resistant starch content in sourdough bread. Higher levels of RS in sourdough products were confirmed also by several other studies, but quantitatively the increases were low and not shown to result in measurable health effects (275, 288–290).

Data on different sourdough additions in tef bread making revealed a clear trend; resistant starch content increased significantly, whereas slowly and rapidly digestible starch decreased with increased sourdough addition to the bread recipe. However, after 5 days of storage, resistant starch values for all recipes were no longer different (291). In contrast, a single study discovered the lowest level of resistant starch in sourdough bread, with lactic acid having only a marginal effect on resistant starch content when compared to yeast-fermented control bread (292).

In summary, all studies with exception of Amaral et al. (292) showed a distinct but small increase in resistant starch in sourdough bread. Confounding factors such as different baking procedures can be excluded because the cited literature applied identical baking conditions (time and temperature) when comparing straight dough and sourdough bread (286). Nonetheless, the storage time may have a significant impact on the resistant starch content (291). The increase in resistant starch may be caused by the presence of acids, which promoted starch retrogradation after heat application during the baking step. However, the degree of gelatinization, porosity and other compounds may also have an impact (3).

### Effect of fermentation on glycemic responses and role of induced bio-acids

Several studies reported a lower GI (3, 275, 285, 293, 294) and an improved insulin response (295) of sourdough bread. Several underlying mechanisms such as the previously discussed resistant starch content, have been proposed (see above). Furthermore, organic acids added to food have been shown to reduce post-ingestion glycemic response and have the potential to reduce the gastric emptying rate and increase meal-induced satiation (285, 296–299). However, using paracetamol adsorption (300) or the 13\u00B0C-octanoic acid breath-test (301) as a marker of gastric emptying, showed no differences in the rate of gastric emptying after ingesting breads made with different ferments. Polese et al. (302) observed that sourdough croissants have a faster gastric emptying when compared to croissants fermented with baker’s yeast only. In contrast, Rizzello et al. (303) showed that gastric emptying after consuming sourdough bread was faster compared to straight dough bread.

In addition to lower blood glucose responses, sourdough bread consumption was linked to lower insulin levels. For example, Juntunen et al. (295) supplied standardized amounts of sourdough (40% pre-dough) rye bread to provide 50 g of bioavailable starch and measured blood and insulin responses in 19 healthy postmenopausal women. The rye breads were made from wholemeal or white rye flour, with varying amounts of added fiber, and they were compared to white wheat bread as a control. The insulin responses were lower in the rye breads. Further, blood glucose responses did not differ between the sourdough rye breads and the control wheat bread, but less insulin was required to regulate blood sugar regardless of bran content. In an additional *in vitro* digestion test, the authors observed that starch hydrolysis was slower in all rye breads than in the white wheat control, and that the structure of the continuous matrix and starch granules differed between the rye and white wheat control breads (295). Apart from bio-acid effects, differences in matrix, starch granule structure, and resistant starch content may explain variations in blood glucose and insulin levels (304–307).

Although it is often suggested that sourdough bread results in a lower glycemic response than yeast fermented bread, data appear to be conflicting. This may result from improper control conditions, in which other variables than only sourdough vs. yeast dough making differ, such as comparing a rye sourdough bread with a yeast wheat bread. In such a comparison also grain type used, flour characteristics, fiber content and bread structure will impact on the glycemic response. Arora et al. (2) compiled data from 22 studies with *in vivo* challenges, and concluded that the average GI of sourdough bread was significantly lower compared to straight dough bread. However, none of these 22 study sources were cited making a critical evaluation of the study conditions and inclusion criteria for combining the results impossible. A recent systematic review and critical analysis of nutritional benefits of sourdough, by Ribet et al. (8), concluded that 50% of medical studies addressing the effects of sourdough fermentation in GI showed no effect, which leaves us to follow the conclusion that an effect of sourdough fermentation on GI is not substantiated.

## Summary and conclusion

The definition of the term “sourdough” as a dough fermented with lactic acid bacteria in addition to yeasts is widely accepted in the scientific literature but there is no established and internationally recognized legal definition of sourdough bread. Descriptions and regulatory definitions differ among countries (158). In the world of artisanal bakers all have their own favorable ‘mother-sourdough’. Apart from commonalities, such as a high level of lactic acid bacteria and higher levels of activity, sourdoughs differ in composition with respect to microbiota subclasses, enzymes and metabolic activity, and the proportion of the flour that is fermented. Most research addressing the effects of sourdough fermentation on compositional changes, used experimental sourdoughs and fermentation conditions, usually leading to significant acidification of the dough to levels of pH < 4.5. Addition of specific microbiota or enzymes (e.g., isolated specific strains of lactobacilli or specific fungal enzymes) leads to a reduction of antinutritive or inflammatory compounds in the dough. However, results from small scale experiments can therefore not be generalized to sourdough breads that are commonly sold. In this respect, analysis of bread samples purchased in the market showed that most of these had pH levels of >5.0, substantially above desired levels of pH < 4.5 to obtain desired changes in composition (158). During several decades of research, compositional changes often suggested to be beneficial for health, such as phytate reduction and increased level of unbound minerals, decrease in FODMAPs and gluten immunogenic peptides, generation of bioactive peptides and increase in free amino acids, etc., in dough and bread, have been reported. Despite these observations, significant health effects on well-defined clinical endpoints such as diabetes risk reduction, improved weight management, reduction of gluten intolerances and improved bone mineral density as a result of sourdough bread consumption have not been clearly shown. Accordingly, there appears to be a clear discrepancy between observations *in vitro/vivo* tests with experimental sourdoughs and supporting data on health effects from human clinical studies. Because of this situation, health authorities such as EFSA (EU) or FDA (United States) have thus so far not approved the use of any sourdough health benefit claim.

Overall, we conclude that although a broad range of sourdough related health benefits are praised in publications, social media and by bakers, a sound evidence base for measurable effects on health related clinical endpoints has not been established. Our conclusions align with those of Ribet et al. (8), who pointed to conflicting evidence and inconsistent results or beneficial effects that appeared not to be significant. Both, sourdough fermentation and prolonged yeast fermentation result in a significant degradation of FODMAPs (range 50–85%) and inclusion of specific microbiota can induce almost complete degradation. Such reductions help mitigate gas formation related intestinal discomfort in IBS patients (8). In combination with raw materials delivering low amounts of harmful proteins, suitable products for patients with wheat related disorders can be produced by sourdough fermentation, e.g., products from oat for CD or einkorn for NCWS. Nevertheless, this conclusion has to be proven by *in vivo* tests and clinical studies. Sourdough fermentation might promote gut microbiota and health as well. However, a recent review pointed out eminent knowledge gaps which limits developments of a personalized nutrition (308). Although this review revealed that sourdough fermentation alone did not result in a direct and measurable health benefit, the sum of changes in structure of ingredients and metabolic products might have indirectly positive effects for human nutrition and wellbeing of consumers. Furthermore, increased shelf life of sourdough bread and reduced susceptibility for spoilage contribute to food safety.

Future sourdough studies should implement appropriate controls, with the only differing variable being the fermentation process (e.g., comparison of wholemeal wheat sourdough vs. wholemeal yeast bread) with identical ingredient recipes. Such studies should also include appropriate analysis of circulating target compounds in the intestine and in required biofluids *in vivo*. The latter is particularly important for determining the effects of potential bioactive peptides. To generalize effects of experimental sourdoughs to those sold in the market, they should have similar composition and processing.

We remain with consistent and reliable evidence that sourdough fermentation improves the sensory and textural quality of bread. Moreover, Sourdough fermentation particularly allows replacement of hyper-palatable baked goods, which are high in sugar, fat, and salt and were shown to increase the *ad libitum* food intake, with more wholesome recipes (309, 310). This beneficial impact on bread quality also relates to superior whole-grain or fiber-enriched products and Thus supports a healthy nutrition and lifestyle.

## Author contributions

VD’A, MG, SD’A, and FB were mainly responsible for conceptualization, LC and HG assisted. VD’A, MG, LC, SD’A, and FB were preparing figures and tables. All authors wrote parts belonging to their expertise, read, and approved the final manuscript.

## Conflict of interest

The authors declare that the research was conducted in the absence of any commercial or financial relationships that could be construed as a potential conflict of interest.

## Publisher’s note

All claims expressed in this article are solely those of the authors and do not necessarily represent those of their affiliated organizations, or those of the publisher, the editors and the reviewers. Any product that may be evaluated in this article, or claim that may be made by its manufacturer, is not guaranteed or endorsed by the publisher.

## Footnotes

^1^ www.allergome.org
